# Meningeal Mast Cells as Key Effectors of Stroke Pathology

**DOI:** 10.3389/fncel.2019.00126

**Published:** 2019-04-03

**Authors:** Ahmet Arac, Michele A. Grimbaldeston, Stephen J. Galli, Tonya M. Bliss, Gary K. Steinberg

**Affiliations:** ^1^Department of Neurology, David Geffen School of Medicine, University of California, Los Angeles, Los Angeles, CA, United States; ^2^OMNI-Biomarker Development, Genentech Inc., South San Francisco, CA, United States; ^3^Department of Pathology, School of Medicine, Stanford University, Stanford, CA, United States; ^4^Department of Microbiology and Immunology, School of Medicine, Stanford University, Stanford, CA, United States; ^5^Department of Neurosurgery, School of Medicine, Stanford University, Stanford, CA, United States; ^6^Stanford Stroke Center, School of Medicine, Stanford University, Stanford, CA, United States

**Keywords:** meninges, mast cells, ischemic stroke, meningeal mast cells, stroke pathology

## Abstract

Stroke is the leading cause of adult disability in the United States. Because post-stroke inflammation is a critical determinant of damage and recovery after stroke, understanding the interplay between the immune system and the brain after stroke holds much promise for therapeutic intervention. An understudied, but important aspect of this interplay is the role of meninges that surround the brain. All blood vessels travel through the meningeal space before entering the brain parenchyma, making the meninges ideally located to act as an immune gatekeeper for the underlying parenchyma. Emerging evidence suggests that the actions of immune cells resident in the meninges are essential for executing this gatekeeper function. Mast cells (MCs), best known as proinflammatory effector cells, are one of the long-term resident immune cells in the meninges. Here, we discuss recent findings in the literature regarding the role of MCs located in the meningeal space and stroke pathology. We review the latest advances in mouse models to investigate the roles of MCs and MC-derived products *in vivo*, and the importance of using these mouse models. We examine the concept of the meninges playing a critical role in brain and immune interactions, reevaluate the perspectives on the key effectors of stroke pathology, and discuss the opportunities and challenges for therapeutic development.

## Introduction

Every year, ~800,000 people suffer from stroke in the United States (Benjamin et al., 2018). The currently available therapies for acute stroke focus on removal of the blood clot, either pharmacologically or mechanically (Fisher and Saver, 2015). With advances in careful patient selection for these therapies, the time window for therapy initiation can be extended up to 24 h (Albers et al., 2018; Nogueira et al., 2018). However, the majority of patients are still not eligible for these therapies. Ischemic stroke occurs when the blood supply to the brain is interrupted by a blood clot. This initiates a cascade of events that includes excitotoxicity, free radical release, mitochondrial changes, and various degrees of immune response that leads to neuronal and glial cell death, blood-brain barrier dysfunction and ultimately the clinical symptoms (Moskowitz et al., 2010; Knowland et al., 2014; George and Steinberg, 2015). The timing of these events differ, and additionally, the post-stroke immune response has different phases that can be detrimental or beneficial (Anrather and Iadecola, 2016). Thus, a detailed understanding of the post-stroke immune response is necessary in order to better utilize its therapeutic potential.

The brain is long considered to be an immune-privileged organ (Louveau et al., 2015a). This is in part due to its anatomical isolation from the rest of the body, having its own unique resident immune cells (microglia), and having restricted access for circulating immune cells under homeostatic conditions. This is important for homeostatic functions but becomes especially important in pathological conditions. More recently, meninges—the membranes surrounding the brain—have been proposed to play important roles in the regulation of brain-immune interactions (Rua and McGavern, 2018). Emerging evidence suggests that the actions of immune cells resident in the meninges are important for the immunoregulatory role of the meninges (Rua and McGavern, 2018). Mast cells (MCs) reside in high numbers within the meninges. These tissue-resident immune cells have proinflammatory and immunoregulatory roles (Grimbaldeston et al., 2007; Biggs et al., 2010; Galli et al., 2011; Tsai et al., 2011), and play key functions in both innate and adaptive immune responses. They act as constitutive or inducible sources of many cytokines, chemokines and proteases (Mukai et al., 2018). This makes them potential targets for therapeutic intervention, depending on their roles in the pathophysiology. Fortunately, there are several methodologies to study the role of MCs and MC-derived products *in vivo* (Galli et al., 2015).

Here, we discuss the evidence that MCs located in the meningeal space can worsen stroke pathology. We also review some of the latest advances in mouse models to investigate the roles of MCs and MC-derived products *in vivo*, and the importance of using these mouse models. We examine the concept of the meninges playing a critical role in brain and immune interactions, reevaluate perspectives on key effectors of stroke pathology, and discuss the opportunities and challenges for therapeutic development.

## Meninges as Key Sites for Brain Immune Access

**Figure 1:**
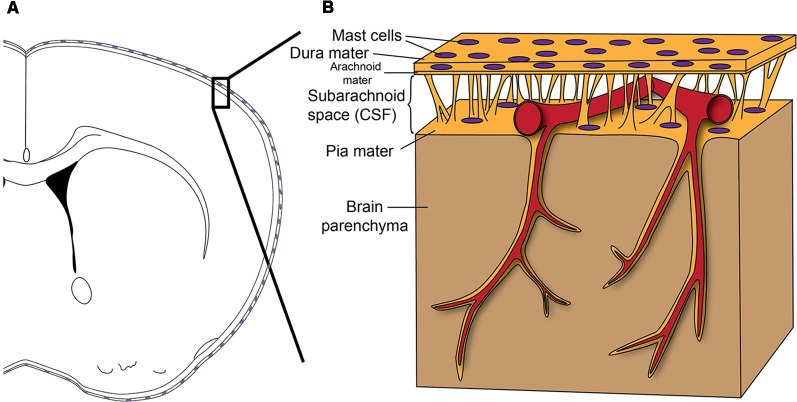
**(A)** Scheme shows how the brain is enveloped by the meninges that contain Mast cells (MCs) in both the dura mater and pia mater. **(B)** Before entering the brain parenchyma, blood vessels course on the surface of the brain between the dura mater and pia mater. Therefore, as a resident immune cell in the meninges, the MC has the potential to influence blood vessels and to function as a gatekeeper to influence brain inflammation and pathology. Reprinted from Arac et al. (2014) with permission from Elsevier.

Meninges are the connective tissue that surrounds the brain and spinal cord. They consist of three layers (Figure 1): an outer thick layer, dura mater, that is attached to the skull, an inner thin layer, pia mater, that is attached to the brain and spinal cord parenchyma, and a spider net-like structure, arachnoid mater, in between the dura and pia maters. The blood vessels and the cerebrospinal fluid exist within the space between the arachnoid mater and pia mater (subarachnoid space). The meninges have long been considered as just an anatomical barrier; however, accumulating evidence suggests that they are important for brain–immune communications in health and disease (Androdias et al., 2010; Derecki et al., 2010; Shechter et al., 2013; Kwong et al., 2017; Benakis et al., 2018). All the arteries that penetrate the brain parenchyma first travel through the subarachnoid space before entering into the brain. The venous blood then travels along the sinuses within the dura mater before exiting the skull. Moreover, the recent discovery of the central nervous system (CNS)’s lymphatic vessels within the meninges highlights their potential role in brain–immune interactions (Aspelund et al., 2015; Louveau et al., 2015b). These meningeal lymphatics have been shown to be important in controlling both neuroinflammatory events and immune cell trafficking (Louveau et al., 2018b). Additionally, direct vascular channels that traverse the meninges have been discovered recently between the bone marrow in the skull and the brain surface (Herisson et al., 2018). These direct vascular channels help enable myeloid cell migration into the brain parenchyma in stroke (Herisson et al., 2018). All these vascular structures (the arteries and veins, meningeal lymphatic vessels, and direct vascular channels) travel through the meninges, making the meninges ideal for a possible gatekeeper role (Rua and McGavern, 2018).

While some immune cells locate to dura mater after stroke (Benakis et al., 2018) or aseptic inflammation (Kwong et al., 2017), there are also tissue-resident immune cells normally residing in the meninges. One such immune cell type is the MC (Dimlich et al., 1991; Arac et al., 2014; Figure 1). MCs are tissue-resident immune cells. Mature MCs do not circulate in the blood but exist especially in tissues exposed to the exterior, such as skin, lungs, and gut (Galli et al., 2011; Tsai et al., 2011). These regions are generally critical for host defense and require strong immune regulation, as they represent the interface between the body and the external environment (Galli et al., 2008). In this respect, it is not surprising that MCs exist at high numbers in meninges as the meninges separate blood and an immune-privileged brain parenchyma (Louveau et al., 2015a). Given the meninges’ potential gatekeeper role in orchestrating brain–immune interactions, meningeal MCs can play significant roles in such interactions.

## Cellular Elements of Post-Stroke Inflammation

Inflammation is critically important in stroke pathology. Several elements of the immune system play roles at different time points after stroke. The first immune cells to respond to interruption of blood flow to the brain are the resident microglia. There is evidence that microglia show signs of activation within 30 min to 1 h after stroke (Ito et al., 2001; Clausen et al., 2008); these changes evolve during the 12 h following stroke and result in decreased microglial numbers within 24 h (Ito et al., 2001). These changes persist several weeks after stroke onset (Lambertsen et al., 2005; Perego et al., 2011). The microglia secrete several pro- and anti-inflammatory cytokines and chemokines (Clausen et al., 2008; Lambertsen et al., 2009), and can be either detrimental (Neher et al., 2013) or beneficial (Lambertsen et al., 2009; Neumann et al., 2009) through secreted factors or direct phagocytosis.

One of the first immune cells that infiltrate the brain after stroke is the neutrophil. They start infiltrating around 6 h after stroke onset and reach peak numbers at 24 h (Perez-de-Puig et al., 2015). The predominant effect of neutrophils appears to be increased ischemic injury as inhibition of their trafficking results in better outcomes (Allen et al., 2012; Jickling et al., 2015; Neumann et al., 2015). However, there is also evidence that neutrophils play role in resolution of inflammation (Cuartero et al., 2013) and promotion of remodeling (Christoffersson et al., 2012) after stroke. Thus, the role of neutrophils in stroke pathology is more complex, which may in part account for the failure of translational approaches targeting neutrophils as stroke therapy (Jickling et al., 2015).

Monocytes follow neutrophils in parenchymal infiltration after a stroke, starting to infiltrate around 6–48 h after stroke onset and staying in the brain weeks thereafter (Lambertsen et al., 2005; Perego et al., 2011). The CCR2^+^Ly6C^high^ monocytes/macrophages are thought to be involved with the acute inflammation, thus worsening the damage, whereas CX3CR1^+^Ly6C^low^ macrophages are considered as the immune cells involved in the repair process (Garcia-Bonilla et al., 2016; Tsuyama et al., 2018). However, monocyte response after stroke is very complicated. For example, the so-called inflammatory CCR2^+^Ly6C^high^ monocytes enter first, then are converted to regulatory CX3CR1^+^Ly6C^low^ macrophages (Garcia-Bonilla et al., 2016). Interestingly, efforts to target these monocytes populations have shown mixed results; reducing monocyte infiltration reduced stroke recovery in one study (Wattananit et al., 2016), while another study concluded that targeting monocyte subsets did not change the outcome after stroke (Schmidt et al., 2017).

The response of lymphocytes (T and B cells) after stroke is quite different from that of the other immune cells as they tend to infiltrate the brain at later time points (days) and stay longer (weeks to months) after stroke onset (Liesz et al., 2013a). The role of these cells depends on the subtype of lymphocytes involved, for example, T cells can worsen the injury (Yilmaz et al., 2006; Clarkson et al., 2014; Mracsko et al., 2014). More specifically, regulatory T cells have been shown to be protective *via* secretion of interleukin-10 (Liesz et al., 2009, 2013b), whereas gamma-delta-T cells can worsen the injury by secreting interleukin-17 (Shichita et al., 2009). Moreover, targeting T cell infiltration by inhibiting their trafficking into the brain parenchyma protected the brain against deleterious neuroinflammation (Liesz et al., 2011b). However, amplifying regulatory T cells with a CD28 superagonist in preclinical studies have elicited conflicting outcomes, and resulted in reduced brain damage in one study (Na et al., 2015), vs. increased brain damage in another (Schuhmann et al., 2015). Moreover, treatment with another immunomodulatory drug, FTY720, despite reducing the post-stroke lymphocyte infiltration after stroke did not improve the post-stroke outcomes (Liesz et al., 2011a). The role of B cells in stroke pathology is also very unclear. Although in some studies B cells did not have any direct effect on the stroke pathology (Yilmaz et al., 2006; Schuhmann et al., 2017), in others they were shown to be beneficial (Ren et al., 2011; Chen et al., 2012). However, B cells were also shown to have delayed deleterious effects such as cognitive impairment after stroke (Doyle et al., 2015).

MCs have been proposed to play important roles in stroke pathology. Specifically, cerebral MCs were shown to worsen the brain swelling and neutrophil accumulation after stroke (Strbian et al., 2006) and stroke induced the degranulation of brain parenchymal MCs (Biran et al., 2008; Jin et al., 2009; Lindsberg et al., 2010). Moreover, a MC stabilizer in rats, cromolyn, was shown to be protective in stroke (Strbian et al., 2007; Jin et al., 2009). Cerebral MCs were also shown to mediate blood-brain barrier disruption after stroke (Mattila et al., 2011; McKittrick et al., 2015). As opposed to these studies, which proposed a role for cerebral MCs in worsening stroke pathology, another study (reviewed in detail below), proposed that meningeal, rather than cerebral, MCs play important roles in exacerbating stroke pathology, in part by secreting IL-6 (Arac et al., 2014).

Overall, despite being a major participant in stroke pathology, inflammatory responses after stroke are complex, and attempts should be made to understand their detailed pathophysiological mechanisms before attempting therapeutic interventions.

## *In Vivo* Models to Study MC Function (“MC Knock-In Mice”)

MCs are tissue-resident immune cells derived from bone marrow. Small numbers of MC progenitors exist in the blood, but they complete their differentiation and maturation in tissue microenvironments (Galli et al., 2008, 2011). MCs can be activated by diverse mechanisms including *via* binding of antigen to antigen-specific IgE (Galli and Tsai, 2012), as well as by physical agents, innate danger signals (Supajatura et al., 2002), venoms (Metz et al., 2006), complement activation (Schäfer et al., 2013), and exposure to certain chemokines and cytokines. Upon activation, MCs can secrete either stored mediators (such as histamine and heparin) or *de novo* synthesized cytokines, chemokines, and growth factors (Mukai et al., 2018). These MC-derived products have been shown to have positive or negative effects on inflammation (Galli et al., 2008). However, many of these MC-derived products are also produced by a variety of other immune cells.

In order to identify a role for MCs in different biological settings, one can ablate MCs selectively (either by a drug or antibody, or genetically) to support their necessity, and then replace their function selectively to support claims of sufficiency. Pharmacological approaches such as MC stabilizers (cromolyn) or activators (c48/80) have commonly been used to infer a role for MCs. However, both of these drugs can have MC-independent effects on other immune cells (Arumugam et al., 2006; Oka et al., 2012; Schemann et al., 2012). There are also other approaches using recombinant MC proteases, such as tryptase, chymase, and tyrosine kinase inhibitors. However, all of these approaches have potential off-target effects limiting the interpretation of the results (Galli et al., 2015).

As these approaches lack true specificity for MCs, genetic approaches represent a more definitive way to assess the functions of MCs *in vivo*. Two commonly used genetically MC-deficient mice are the *Kit*^W/W-v^ and *Kit*^W-sh/W-sh^ mice. These mice have different types of mutations affecting the c-*kit* gene that result in a profound deficiency of MCs and melanocytes in both mice. However, both WBB6F_1_-*Kit*^W/W-v^ and C57BL/6-*Kit*^W-sh/W-sh^ also have several other abnormalities within and outside the immune system, including effects on hematopoietic cells other than MCs (Galli et al., 2015). Thus, the differences in the biological responses in these *Kit*^W/W-v^ and *Kit*^W-sh/W-sh^ mice compared with wild type (WT) mice may, in principle, reflect any of the abnormalities in these mice and are not necessarily due to their MC deficiency. However, the MC deficiency in these mice can be selectively “repaired” by the adoptive transfer of *in vitro*-derived WT or mutant MCs (Nakano et al., 1985; Galli et al., 2005; Grimbaldeston et al., 2005). These *in vitro*-grown, bone marrow-derived, cultured MCs (BMCMCs) can be administered systemically or locally to create the so-called “MC knock-in mice” (Galli et al., 2015). These engrafted BMCMCs were shown to survive, and function normally, up to 18 weeks in lungs (Yu et al., 2006), 12 weeks in the skin (Biggs et al., 2010), and 10 weeks in the meninges (Arac et al., 2014). Moreover, the numbers of MCs in the MC knock-in mice are generally equivalent to those of the WT animals (Yu et al., 2006; Biggs et al., 2010; Arac et al., 2014). MC knock-in mice have been widely used to assess the roles of MCs in several biological events *in vivo*.

Despite the power of MC knock-in mice, these mice still carry potential problems inherent to c-*kit* related abnormalities (and other abnormalities in the *Kit*^W-sh/W-sh^ mice) in the mutant mice. To overcome this issue, MC-deficient mice with normal c-*kit* function were developed by using MC-specific Cre recombinase approach (Dudeck et al., 2011; Feyerabend et al., 2011; Lilla et al., 2011). Such mice profoundly lack MCs (and to a lesser extent, basophils), but have normal c-*kit* function and lack the known abnormalities that the c-*kit* mutant mice have. Moreover, inducible models of MC deficiency were also developed by using the MC-specific Cre recombinase and inducible diphtheria toxin receptor expression (Dudeck et al., 2011; Otsuka et al., 2011; Reber et al., 2014). Injection of diphtheria toxin locally to the site of interest results in profound depletion of local MCs in these mice.

Given all these *in vivo* models to study MC function (with different pros and cons), one recommended approach has been to use more than one of these models to assess the initial biological responses, and if there are consistent results between the models, then proceeding to further studies (Galli et al., 2015). For additional discussion of these models, we recommend reading this review (Galli et al., 2015).

## Meningeal MCs Exacerbate Stroke Pathology

We utilized some of these various *in vivo* models in order to assess the role of MCs in stroke pathology (Arac et al., 2014). WBB6F_1_-*Kit*^W/W-v^ mice had significantly smaller infarcts and less brain swelling compared to WT controls at 3 and 14 days after stroke. Systemic engraftment of WT MCs in these c-*kit* mutant mice resulted in the same extent of injury as the WT mice. In parallel to these results, the MC-deficient *Cpa3-Cre; Mc1–1*^fl/fl^ mice (Lilla et al., 2011; MC-deficient mice with normal c-*kit* function) also had a smaller extent of stroke pathology when compared to their corresponding WT counterparts. Both mouse models also showed a similar MC-dependent pattern for myeloid, but not lymphoid, cell numbers in the brain at 3 days after stroke. Together, these data from two different types of *in vivo* mouse models of genetically-determined MC deficiency provide strong support that MCs play an important role in worsening the brain injury after stroke.

**Figure 2:**
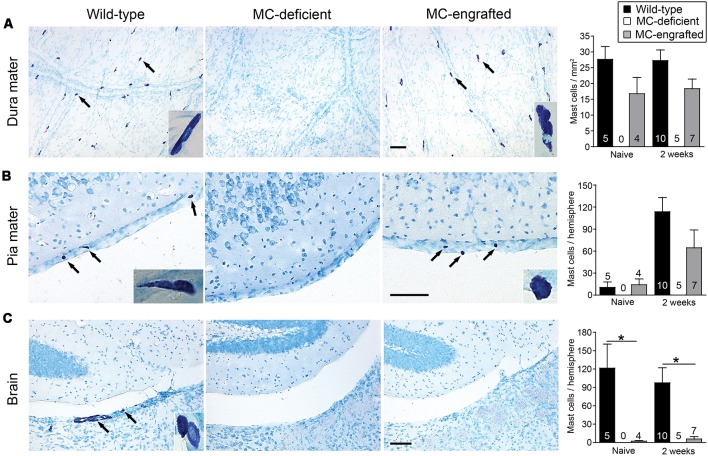
Location of MCs in the central nervous system (CNS). Representative toluidine blue-stained images of and quantification of MCs in dura mater **(A)**, pia mater **(B)**, and brain parenchyma **(C)** of MC-deficient WBB6F1-*Kit*^W/W-v^ mice and the corresponding wild type (WT) mice and MC-engrafted *Kit*^W/W-v^ mice. MCs stain purple with toluidine blue; arrows indicate representative MCs. Insets show magnified images of MCs in each tissue. Data are expressed as means+SEM. The number of mice in each group is indicated in (or over) each bar. *P < 0.05. Scale bars: 100 mm **(A–C)**. Reprinted from Arac et al. (2014) with permission from Elsevier.

Because the MC deficiencies in both of these models are in all examined tissues, it is hard to discern which population of MCs might be critical for this effect. Thus, we compared the number of CNS MCs in the MC-engrafted WBB6F_1_-*Kit*^W/W-v^ mice to those of WT (WBB6F_1_-*Kit*^+/+^) mice. Both WT mice and MC-engrafted mice had similar numbers of MCs in the dura and pia mater both before and 2 weeks after stroke (Figures 2A,B). In contrast, the brain parenchyma of the MC-engrafted mice had either no or substantially fewer MCs (Figure 2C). These data strongly suggest that brain parenchymal MCs are not responsible for the MC-dependent worsening of the stroke pathology observed in this mouse model. It instead suggests a potential role for meningeal MCs in modulating this response. Moreover, the density of MCs in the dura mater of both WT and MC-engrafted mice (15–27 cells/mm^2^) is similar to that in humans (Varatharaj et al., 2012; 11–23 cells/mm^2^). In order to calculate these dural MC densities, we used dura mater whole mount preparations (Figure 2A). This whole mount preparation of dura mater was also later used to identify the meningeal lymphatics (Louveau et al., 2015b, 2018a).

**Figure 3:**
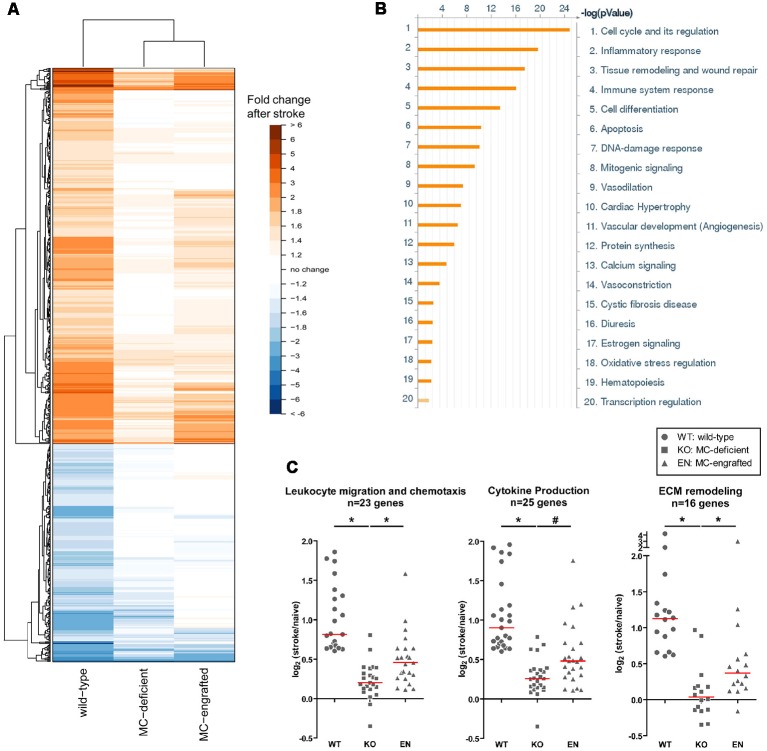
Meningeal gene expression changes after stroke. **(A)** Heatmap showing the fold-change in expression of genes significantly changed (*p* < 0.05) after stroke in WT (WBB6F_1_-*Kit*^+/+^) mice with the corresponding change in expression in MC-deficient (WBB6F_1_-*Kit*^W/W-v^) and MC-engrafted [WBB6F_1_-*Kit*^W/W-v^ mice which had been engrafted in the meninges with WT bone marrow-derived, culturedMCs (BMCMCs)] mice (irrespective of whether the gene changes in these two groups after stroke reach significance). The genes represented are those with an absolute fold-change after stroke of ≥1.5 in WT mice. **(B)** Pathway map of the functional grouping of genes that were significantly upregulated after stroke in WT mice. **(C)** Dot plots showing the log_2_ fold-change in expression after stroke of genes within the indicated gene ontology groups in each of three mouse groups. The genes represented in each gene ontology group were significantly upregulated after stroke in WT mice, with a fold-change of ≥1.5. Each data point within a mouse group represents a different gene. Red line indicates median. *p < 0.05; ^#^*p* = 0.05.

To test whether the meningeal MCs are sufficient in modulating the MC-dependent stroke pathology, we engrafted BMCMCs locally into the meninges. With a modified intracranial injection method, the MCs engraft only in the meninges and the number of meningeal MCs in MC-engrafted animals are similar to those of the WT mice (Sayed et al., 2010; Arac et al., 2014). After meningeal engraftment of MCs, MC-engrafted mice developed significantly worse injury after stroke (Arac et al., 2014). This provides strong evidence that the meningeal MCs are sufficient to elicit the MC-dependent effects in stroke pathology. Moreover, we found by microarray analysis of the dura of MC-deficient WBB6F_1_-*Kit*^W/W-v^ mice, the corresponding WT (WBB6F_1_-*Kit*^+/+^) mice, and WBB6F^1^-*Kit*^W/W-v^ mice which had been engrafted in the meninges with WT BMCMCs (unpublished data), that the meninges are a site of inflammation-related activity after stroke and that the meningeal inflammatory gene response to stroke is modulated, at least in part, by MCs (Figure 3A). We found that many stroke-activated genes in the meninges are involved in the regulation of inflammatory and immune system processes. These include cytokines, chemokines, cell adhesion molecules, immune signaling receptors and extracellular matrix remodeling molecules, all of which are known to contribute to leukocyte migration and activation (Figure 3B). Notably, we found that many of the stroke-induced gene changes involved in inflammation were either absent or exhibited a smaller response in MC-deficient mice, but were recapitulated when MCs were engrafted into these mice, consistent with the idea that MCs can modulate the meningeal inflammatory gene changes after stroke (Figure 3C).

In order to identify the MC-derived products that influence stroke pathology, BMCMCs derived from two candidate factor-deficient mice (IL-6 and CCL7) were injected into the meninges of MC-deficient mice (Arac et al., 2014). We found that mice meningeally engrafted with BMCMCs which lack IL-6 failed to demonstrate MC-dependent responses in stroke pathology compared to the WT BMCMC engrafted mice. By contrast, MC-derived CCL7 had less of an effect on stroke pathology (Arac et al., 2014). This demonstrates how meningeal MC-derived IL-6 can, in part, explain the MC-dependent effects in stroke pathology.

There are several questions that require further studies. For example, how does the meningeal MC-derived IL-6 execute its function to exacerbate stroke pathology? What downstream pathways are involved in this biological response? Does it alter the meningeal access of immune cells to the brain? What are its effects on brain meningeal lymphatics? Likewise, what are the mechanisms involved in stimulating the meningeal MCs to release IL-6 after a stroke? Are there other MC-derived factors that also might be involved in worsening the stroke pathology? Contrary to these, could anti-coagulative effects of MC-derived heparin proteoglycan and/or proteases have effects in dissolving the blood clot in ischemic stroke? Moreover, there could also be other, yet to be identified, meningeal MC-derived products that are important in the repair process after stroke. Thus, one should be cautious in directly targeting MCs as a therapeutic for stroke (Ocak et al., 2018). These and many other questions need to be investigated to better understand the role of meningeal MCs in stroke pathology.

## Opportunities and Challenges for Future

Immune responses after stroke are complex with many elements involved, and it is not the focus of this article to comprehensively review that literature; interested readers can find up-to-date review articles on this topic (McCombe and Read, 2008; Iadecola and Anrather, 2011; Macrez et al., 2011; Lambertsen et al., 2018; Rayasam et al., 2018). The post-stroke immune response potentially holds very promising targets for therapeutic interventions for stroke (Arac et al., 2011; Macrez et al., 2011). However, these post-stroke immune events have not been well characterized, with many of the immune responses potentially having dual roles. Development of new and effective therapeutics for stroke will require detailed, mechanistic studies before consideration of specific approaches for therapeutic intervention. Here, we have discussed recent advances in post-stroke immune response with a particular focus on the role of meningeal MCs. Given the increasing importance and key roles of meninges in the regulation of brain–immune interactions (Rua and McGavern, 2018), meningeal MCs are ideally located to play potentially key roles in these interactions. As MCs have diverse roles in many immune responses (Galli et al., 2005, 2008), their key location in the meninges makes them potentially important players in the regulation of immune responses in the brain during health and disease.

Identifying the roles of several immune elements in stroke pathology is critical for the development of effective therapeutics. Fortunately, there are many advanced immunological tools to perform mechanistic studies. Without such detailed characterization of post-stroke immune responses, performing clinical tests of potential targets will likely result in failures, and may decrease the enthusiasm for future studies.

## Data Availability

The datasets generated for this study are available on request to the corresponding author.

## Author Contributions

AA wrote the initial draft of the review. All authors contributed to revising the manuscript, reading and approving the submitted version.

## Conflict of Interest Statement

GS is a consultant for Qool Therapeutics, Peter Lazic US, Inc., and NeuroSave. MG is employed by Genentech Inc.

The remaining authors declare that the research was conducted in the absence of any commercial or financial relationships that could be construed as a potential conflict of interest.
